# The multifaceted role of oral microbiota-host interactions in oral submucous fibrosis: from pathogenic mechanisms to microecological regulation therapies

**DOI:** 10.3389/fmicb.2026.1901183

**Published:** 2026-07-30

**Authors:** Yan Xie, Saifei Xie, Mengying Shao, Lizhen Zhuang, Taohua Pan, Shun Mao, Hui Xie

**Affiliations:** 1 School of Stomatology, Hunan University of Chinese Medicine, Changsha, China; 2 Department of Periodontal and Oral Mucosa, Changsha Stomatological Hospital, Changsha, China; 3 College of Integrated Traditional Chinese and Western Medicine, Hunan University of Chinese Medicine, Changsha, China

**Keywords:** betel quid, microbiota-host interaction, microecological modulation, oral microbiota, oral submucous fibrosis

## Abstract

Oral submucous fibrosis (OSF) is a precancerous condition closely associated with areca nut chewing, yet its pathogenesis remains incompletely elucidated. Recent studies have highlighted the critical role of oral microbiota dysbiosis in the progression of OSF. This article systematically reviews alterations in the oral microbiota spectrum associated with OSF, the synergistic pathogenic mechanisms between microbiota and areca nut, core pathways of microbiota-host interactions, and therapeutic strategies based on microbiota modulation. Areca nut chewing modifies the oral microenvironment, suppressing commensal bacteria while enriching pathogens. In OSF patients, pathogenic bacteria such as *Fusobacterium nucleatum*, *Porphyromonas gingivalis*, and *Candida* species are significantly enriched, whereas beneficial bacteria including *Lactobacillu*s and *Streptococcus* species are reduced. Microbiota dysbiosis promotes fibrogenesis through multiple mechanisms, including disruption of the mucosal epithelial barrier, activation of TLR/NF-κB and NLRP3 inflammatory pathways, induction of Th17/Treg imbalance, interference with tryptophan and short-chain fatty acid metabolism, and exacerbation of oxidative stress. Microbiota-based therapeutic approaches include probiotics, specific targeted antimicrobial peptides, photodynamic therapy, oral microbiota transplantation, and dietary intervention. Future studies should employ multi-omics integrative analyses to establish causality between microbiota dysbiosis and OSF and to advance the clinical translation of precision microbiota-based therapies.

## 1 Introduction

Oral submucous fibrosis (OSF) is a chronic, progressive oral mucosal disorder characterized by aberrant collagen metabolism and progressive fibrosis of the submucosal layer, leading to mucosal rigidity, loss of elasticity, and functional impairment (Chhabra et al., 2023). OSF is associated with a malignant transformation rate of approximately 4.2% (Chen et al., 2023) and is classified by the World Health Organization as an oral potentially malignant disorder. The development of OSF is strongly associated with betel quid chewing, which is considered the primary etiological factor (Mehrtash et al., 2017). In addition, accumulating evidence suggests that genetic susceptibility, immune dysregulation, and nutritional deficiencies may also contribute to disease onset and progression (Chhabra et al., 2023). Epidemiologically, OSF affects approximately 25 million individuals worldwide, with a high prevalence in South and Southeast Asia, including India, Southern China, and Taiwan (Qin et al., 2023), and it predominantly occurs in individuals aged 20 to 40 years (Shih et al., 2019). Notably, in Hunan Province, China, the incidence of areca nut-related oral cancer has increased rapidly over the past decade, with projections exceeding 100,000 cases by 2030 (Hu et al., 2017; Shao et al., 2026). Therefore, OSF represents a significant global health burden, and its diagnosis, prevention, and management remain critical clinical challenges.

The oral cavity harbors one of the most diverse microbial ecosystems in the human body, comprising bacteria, fungi, viruses, archaea, and bacteriophages. Distinct ecological niches across oral surfaces—including the gingiva, buccal mucosa, palate, tongue, and saliva—shape a spatially heterogeneous microbiota that contributes to immune regulation, mucosal barrier integrity, and oral homeostasis (Lamont et al., 2018; Deo and Deshmukh, 2019). Disruption of oral ecological balance results in oral dysbiosis, characterized by reduced microbial diversity, enrichment of pathogenic taxa, and functional alterations (Talapko et al., 2024). Dysbiosis may impair mucosal barrier integrity and immune homeostasis, contributing to the development of oral potentially malignant disorders (OPMDs). Analysis of biopsy samples from patients with oral leukoplakia (OL), proliferative verrucous leukoplakia (PVL), oral squamous cell carcinoma (OSCC), and PVL progressing to OSCC revealed reduced microbial diversity and enrichment of pathogenic taxa, with microbial diversity being significantly lower in OSCC and PVL-to-OSCC than in healthy controls (Herreros-Pomares et al., 2023). Consistently, a salivary microbiome study reported stage-dependent alterations in microbial diversity and community composition across OL, PVL, and OSCC, while beta diversity analysis revealed distinct microbial clustering among different disease stages (Intini et al., 2025). Taken together, these findings suggest that progressive oral dysbiosis accompanies the transition from OPMDs to OSCC, supporting a potential role for microbiota alterations in disease progression and malignant transformation.

As a representative OPMD, OSF has attracted considerable attention because of its strong association with betel quid chewing and high risk of malignant transformation. Emerging evidence suggests that oral dysbiosis plays an important role in OSF pathogenesis. A clinical study comparing OSF and OSF-associated OSCC demonstrated progressive microbial dysbiosis during malignant transformation, characterized by reduced microbial diversity, disrupted co-occurrence networks, and altered functional metabolic potential (Chen et al., 2021). Betel quid chewing induces persistent alterations in the oral microenvironment that contribute to dysbiosis in OSF (Xiong et al., 2018; Bahuguna et al., 2023). This dysbiosis impairs mucosal immune defense and epithelial homeostasis, thereby promoting pathological tissue remodeling characteristic of OSF (Hernandez et al., 2017). Moreover, oral dysbiosis may contribute to OSF progression through both local and systemic mechanisms. Alterations in the local microbial community disrupt the oral mucosal barrier, promoting pro-inflammatory cytokine release and chronic inflammation (Huang et al., 2023), and may further facilitate microbial translocation along the oral-gut axis, leading to intestinal barrier dysfunction and systemic immune alterations that may contribute to OSF progression (Suarez et al., 2021).

Despite growing evidence linking oral microbiota dysbiosis to OPMDs and OSF, the mechanisms underlying microbiota-host interactions in OSF have not yet been systematically integrated. Therefore, elucidating the mechanisms underlying oral microbiota-host interactions and advancing research on targeted modulation of the oral microbiota are of critical importance for the prevention and treatment of OSF. This article aims to review the pathological relationship between dysbiosis and OSF, outline key mechanisms of microbiota-host crosstalk, and summarize recent advances in microbiota-based therapeutic strategies, thereby providing a clear framework for future research and clinical intervention in OSF.

## 2 The pathological relationship between microbial dysbiosis and OSF

### 2.1 Enrichment of pathogenic microorganisms

The oral microbial profile of OSF patients differs significantly from that of healthy individuals, characterized notably by an enrichment of periodontal pathogens, which may contribute to chronic inflammation-driven mucosal fibrosis (Li et al., 2025). Studies have shown a marked increase in the abundance of *Fusobacterium nucleatum (F. nucleatum)* and *Porphyromonas gingivalis (P. gingivalis)* in OSF patients (Curtis et al., 2020; Kamio et al., 2025), and this microbial shift is positively correlated with the severity of chronic mucosal inflammation.

*F. nucleatum* contributes to mucosal damage primarily through epithelial invasion and the mediation of chronic inflammation and immune dysregulation. Facilitated by its virulence factors, *F. nucleatum* adheres to and invades epithelial cells via the following mechanisms:① The autotransporter protein Fap2 facilitates bacterial adhesion by binding to galactose-galactosamine (Gal-GalNAc) receptors on the host cell surface (Pignatelli et al., 2023); ② The adhesin FadA binds to E-cadherin on epithelial cells, disrupting intercellular junctions and enhancing invasive capability (Meng et al., 2021); ③ The outer membrane protein FomA promotes bacterial colonization and activates host inflammatory responses (Zhang Z. et al., 2022). Furthermore, *F. nucleatum* can suppress caspase activity and upregulate anti-apoptotic proteins such as Bcl-2, thereby inhibiting apoptosis in infected cells and promoting the proliferation of cancer cells (Chattopadhyay et al., 2019). This process indirectly induces epithelial damage, ultimately compromising the mucosal barrier and impairing its ability to resist inflammation. *F. nucleatum* can also stimulate TLR2 and TLR4 receptors, activating the NF-κB pathway and triggering the substantial release of pro-inflammatory cytokines such as IL-6, TNF-*α*, and IL-1β (Bashir et al., 2016). Additionally, it mediates NLRP3 inflammasome activation, leading to caspase-1 cleavage and promoting the maturation and release of IL-1β, thereby amplifying the inflammatory cascade (Sun et al., 2024). Moreover, *F. nucleatum* utilizes its autotransporter protein Fap2 to engage the T cell immunoreceptor with Ig and ITIM domains (TIGIT), thereby inhibiting natural killer (NK) cell function and compromising antitumor immune responses (Gur et al., 2015).

Similarly, P*. gingivalis* can induce epithelial damage and mediate immune evasion and chronic inflammation through the action of its virulence factors. The surface of *P. gingivalis* is covered with two types of pili (long and short), which contain the structural proteins FimA and Mfa1. These proteins bind to host cell receptors such as β1-integrin and GAPDH, triggering actin cytoskeleton reorganization and facilitating bacterial internalization (Marcano et al., 2021; Tortora et al., 2023). *P. gingivalis* also secretes proteolytic enzymes, including gingipains—specifically arginine-gingipain and lysine-gingipain—which degrade intercellular junction proteins such as E-cadherin and occludin (Olsen, 2021). This proteolytic activity disrupts epithelial barrier integrity, facilitates bacterial invasion via the paracellular pathway, and specifically cleaves adhesion molecules like N-cadherin, further promoting pathogen dissemination and tissue damage. Additionally, gingipains and LPS of *P. gingivalis* can activate caspases and receptor-interacting protein kinase 3 (RIPK3), thereby triggering cascade apoptotic pathways (Geng et al., 2022). Notably, studies have revealed that *P. gingivalis* can suppress glutathione peroxidase 4 (GPX4)-mediated anti-ferroptotic activity, leading to lipid peroxidation and mitochondrial shrinkage. This process ultimately induces epithelial cell death and compromises intercellular junctions (Shi et al., 2024). In terms of immune evasion and sustained chronic inflammation, *P. gingivalis* employs the following three mechanisms:

1. Complement System Interference: Secretes gingipains that cleave C5, activating the C5aR-CXCR4 axis to stimulate PKA, inhibiting NF-κB and attenuating antibacterial responses. Alternatively, directly hydrolyzes C3 and C5a, impairing opsonophagocytosis (Seers et al., 2020).
2. Suppression of Interferon Signaling: Degrades IFN receptors and STAT1, blocks interferon-stimulated gene (ISG) expression, and induces TLR2 promoter methylation to reduce receptor expression (Lamont et al., 2023).
3. Inflammatory Polarization Shift: Induces APCs to secrete IL-1β, IL-6, and IL-23, promoting Th17 differentiation and driving chronic tissue destruction (Moutsopoulos et al., 2012).

Furthermore, studies indicate a synergistic interaction between *F. nucleatum* and *P. gingivalis*. Co-culture significantly upregulates the expression of *P. gingivalis* virulence factors, and their crosstalk exacerbates damage to the oral mucosa (Lee et al., 2018).

In addition to *F. nucleatum* and *P. gingivalis*, multiple case–control studies have demonstrated a significantly higher oral *Candida* carriage rate in patients with OSF compared to healthy individuals (Redhu et al., 2021). The colonization rate correlates closely with disease severity, being particularly elevated in advanced OSF cases (Patel et al., 2022). This increase is attributed to pronounced mucosal atrophy, accelerated fibrosis, and salivary gland dysfunction in late-stage disease, while betel quid chewing may further contribute to reduced salivary pH, ultimately leading to decreased salivary flow and reduced levels of antimicrobial components, thereby disrupting oral microbial homeostasis. (Gupta et al., 2015). C*andida*-secreted proteases and phospholipases may exacerbate mucosal epithelial atrophy and act synergistically with TGF-*β*-mediated fibrotic processes to promote abnormal epithelial hyperplasia (Redhu et al., 2021). Mechanistically, *Candida*-derived secreted aspartyl proteases (SAPs) degrade epithelial adhesion molecules such as E-cadherin, leading to disruption of intercellular junctions and epithelial atrophy (Richardson et al., 2018), while phospholipases directly damage host cell membranes and induce epithelial cell lysis. Moreover, candidalysin-mediated epithelial injury induces sustained inflammation. Persistent Candida infection may trigger repeated epithelial injury-repair cycles (Moyes et al., 2016), during which EGFR-and MAPK-related signaling supports tissue repair but may also promote aberrant epithelial proliferation and hyperplastic changes (Sukmana et al., 2024). Based on the available evidence, *Candida* may reasonably be considered an opportunistic pathogen in OSF, although it has not been established as an independent causative factor in OSF malignant transformation.

Although most OSF microbiome studies have focused on bacteria and fungi, limited evidence has also explored the potential involvement of viruses and other non-bacterial microorganisms. A study analyzing OSMF tissues reported detectable high-risk human papillomavirus (HPV) infection. Among 208 patients, HPV-16 E6 DNA was identified in approximately 26–27.4% of cases, suggesting a potential association between HPV infection and OSMF (Chaudhary et al., 2010). However, evidence regarding viruses and other non-bacterial microorganisms in OSF remains limited and is largely restricted to association-based findings, with no well-defined mechanistic studies elucidating their roles in disease pathogenesis.

### 2.2 Depletion of probiotics

In addition to the enrichment of pathogenic bacteria, a reduction in the abundance and diversity of probiotic species represents another characteristic of microbial alterations in OSF patients. This is primarily manifested as a significant decrease in commensal probiotics such as *Lactobacillus, Streptococcus, Veillonella,* and *Bifidobacterium* (Devine et al., 2015; Homayouni Rad et al., 2023). Probiotics play a crucial role in maintaining oral microecological homeostasis by competitively inhibiting pathogen growth, secreting antimicrobial substances, and modulating immune responses to protect the host (Wang et al., 2021). Taking *Lactobacillus* as an example, its core mechanisms in regulating oral ecological balance include pathogen inhibition, competitive exclusion, and immune modulation. *Lactobacillus* not only secretes lactic acid to lower oral pH, thereby suppressing the growth of acid-sensitive pathogens (Boisen et al., 2023), but also produces antimicrobial compounds such as hydrogen peroxide and bacteriocins, which disrupt pathogen membranes or directly kill pathogenic bacteria (Wasfi et al., 2018). In terms of competitive exclusion, Lactobacillus competes with pathogens for adhesion sites and carbon sources, reducing their colonization. Simultaneously, it secretes signaling molecules to interfere with bacterial quorum sensing systems, inhibiting the expression of virulence genes in pathogens (Wu et al., 2022). Regarding immune regulation, Lactobacillus promotes M1 macrophage polarization to enhance pathogen clearance (Duan B. et al., 2022), downregulates pro-inflammatory cytokines including IL-6, IL-8, and TNF-*α* to alleviate inflammation, and stimulates mucin production by epithelial cells, thereby strengthening the mucosal physical barrier (Dempsey and Corr, 2022).

Moreover, *Lactobacillus* exhibits synergistic effects with other probiotics. Studies have shown that *Lactobacillus* and *Streptococcus salivarius* coexist synergistically to inhibit *Candida albicans* proliferation (Salari and Ghasemi Nejad Almani, 2020). When co-cultured with *Streptococcus thermophilus*, lactic acid production is synergistically enhanced, leading to significantly stronger antimicrobial effects. Consequently, the depletion of probiotics weakens these protective mechanisms, creating favorable conditions for pathogen colonization and expansion (Table 1).

**Table 1:** Alteration in oral microbiota, underlying mechanisms, and key effects in OSF.

Microbiota alteration	Representative taxa	Primary mechanisms/virulence factors	Key effects/consequences	References
Pathobionts	*Fusobacterium nucleatum*	Epithelial invasion and disruption: • Fap2, FadA, FomA-mediated adhesion/invasion • Caspase inhibition; •Bcl-2 upregulation Mediation of Inflammation and immunity: • TLR2/TLR4–NF-κB activation • NLRP3 inflammasome activation • Fap2–TIGIT interaction (NK cell inhibition)	• Epithelial barrier disruption • Pro-inflammatory cytokine;production (IL-6, TNF-*α*, IL-1*β*) • Chronic inflammation • Anti-tumor immune suppression	Pignatelli et al. (2023); Meng et al. (2021); Zhang Z. et al. (2022); Chattopadhyay et al. (2019); Bashir et al. (2016); Sun et al. (2024); Gur et al. (2015);
	*Porphyromonas gingivalis*	Epithelial damage: • FimA/Mfa1-mediated adhesion and invasion • Gingipain-mediated barrier disruption (Rgp/Kgp) • Apoptosis and ferroptosis induction Immune evasion and Chronic inflammation: • Complement evasion (C3/C5 cleavage) • Interferon signaling suppression • IL-1β production and Th17 polarization	• Epithelial barrier disruption • Exacerbated mucosal injury (synergistic with *Fusobacterium nucleatum*) • Impaired antibacterial immunity • Chronic inflammation and tissue destruction	Marcano et al. (2021); Tortora et al. (2023); Olsen (2021); Geng et al. (2022); Shi et al. (2024); Seers et al. (2020); Lamont et al. (2023); Moutsopoulos et al. (2012);
	*Candida* spp.	• Opportunistic colonization (mucosal atrophy, hyposalivation, altered salivary pH) • Protease and phospholipase secretion	• Synergy with TGF-β signaling • Epithelial dysplasia promotion • Non-independent etiological factor	Redhu et al. (2021); Patel et al. (2022); Gupta et al. (2015); Redhu et al. (2021); Richardson et al. (2018); Moyes et al. (2016); Sukmana et al. (2024);
Commensals /probiotics (depleted)	*Lactobacillu;* *Streptococcus;* *Veillonella;* *Bifidobacterium spp. and other probiotics*	• Antimicrobial activity: Lactic acid, H₂O₂, and bacteriocin production • Competitive exclusion: Adhesion-site competition and quorum sensing interference • Immunomodulation: M1 macrophage polarization; IL-6/IL-8 suppression • Barrier enhancement: Mucin secretion • Anti-Candida synergy: Cooperation with *Streptococcus salivarius*	• Loss of colonization resistance • Pathogen overgrowth • Impaired immune regulation and barrier function	Devine et al. (2015); Homayouni Rad et al. (2023); Wang et al. (2021); Boisen et al. (2023); Wasfi et al. (2018); Wu et al. (2022); Duan B. et al. (2022); Dempsey and Corr (2022) Salari and Ghasemi Nejad Almani (2020)

### 2.3 Interaction between betel quid chewing and oral microbiota

The main chemical components in betel quid include alkaloids, phenolic compounds, and tannins (Tong et al., 2024). Arecoline have been shown to induce cytotoxicity, genotoxicity, and oxidative stress in oral epithelial cells, promote fibroblast activation, and enhance extracellular matrix deposition, thereby contributing to the development of OSF and increasing malignant transformation risk (Kondaiah et al., 2019). In addition to the host-mediated effects, betel quid components significantly impact the composition and function of the oral microbiota through multiple pathways. Phenolics and tannins can disrupt the integrity of bacterial cell membranes, inhibit glycolytic enzymes to reduce acid production, and selectively suppress acid-sensitive probiotics while promoting the proliferation of acid-tolerant pathogens (Bahuguna et al., 2023). Arecoline, on the other hand, competitively inhibits respiratory chain enzymes in bacteria, suppresses ATP synthesis, and disrupts energy metabolism, thereby inhibiting the growth of commensal bacteria (Chen et al., 2022). Arecoline can also disturb the oral acid–base balance and exerts a dynamic influence on salivary pH. In the initial stage of betel quid chewing, pH may transiently increase due to continuous saliva secretion stimulated by chewing and the alkalinity of arecoline itself (Donoghue et al., 2015). This creates an alkaline environment favorable for the growth of anaerobic pathogenic bacteria while inhibiting beneficial bacteria (Zorba et al., 2021). However, long-term betel quid chewers experience a decline in salivary flow rate and buffering capacity due to the effect of arecoline on the autonomic nervous system, leading to a sustained decrease in pH (Jain et al., 2023), which weakens the physical clearance of pathogenic microbes. Furthermore, betel quid chewing can induce disruption of microbial biofilms. One study indicated that arecoline can inhibit biofilm formation of cariogenic bacteria such as *Bacillus gaemokensis* via hydrophobic interactions that disrupt phospholipid layers. However, long-term use damages the structure of normal microbial biofilms, thereby weakening their protective effect on the mucosa (Jalil et al., 2022). Under the influence of arecoline, changes also occur in microbial metabolites—for instance, increased bacterial production of acetaldehyde, which damages epithelial cell DNA (Pignatelli et al., 2022), which is closely associated with the malignant transformation of OSF.

## 3 Core mechanisms of microbiota-host interactions

### 3.1 Mucosal barrier disruption

Oral dysbiosis contributes to the pathogenesis and progression of OSF through multiple mechanisms. Products secreted by pathogenic bacteria can physically disrupt the epithelial structure. Gingipains, proteases produced by *P.gingivalis*, degrade epithelial junctional proteins: the Rgp fragment specifically cleaves N-cadherin, E-cadherin, and VE-cadherin, thereby disrupting adherens junctions and weakening intercellular mechanical integrity (Sheets et al., 2008). Meanwhile, the Kgp fragment targets junctional adhesion molecule-1 (JAM-1) and the CXADR protein, impairing tight junctions and increasing epithelial barrier permeability (Takeuchi et al., 2022). Additionally, gingipains can degrade integrins, compromising cell anchorage to the basement membrane and leading to epithelial desquamation (Sheets et al., 2008). On the other hand, gingipains also disrupt basement membrane and extracellular matrix (ECM) components (Ruggiero et al., 2013). For instance, they damage the cell-binding domain of fibronectin, depriving cells of ECM attachment sites; degrade tenascin-C, inhibiting fibroblast adhesion and migration thereby compromising tissue repair capacity; and break down type IV collagen and laminin, disrupting basement membrane integrity and further facilitating bacterial penetration. Upon detachment from the ECM, epithelial cells may undergo anoikis. One *in vitro* study demonstrated that after gingipain-mediated degradation of fibronectin, the epithelial cell desquamation rate increased by 300% (Hocevar et al., 2020). Apoptosis of epithelial cells further compromises the mucosal barrier.

*F. nucleatum* disrupts the oral mucosal barrier through mechanisms similar to those of *P.gingivalis*. Its secreted virulence factor, the adhesin FadA, binds to E-cadherin, leading to loss of E-cadherin activity and weakening intercellular adhesion (Lin et al., 2021). Meanwhile, invasins such as the Fap2 outer membrane protein and FomA further promote bacterial adhesion to the epithelial surface and colonization. Through internalization, these proteins facilitate bacterial invasion into cells, thereby disrupting epithelial integrity (McIlvanna et al., 2021).

### 3.2 Immune activation

In terms of immune regulation, microbial dysbiosis can lead to aberrant activation of pattern recognition receptors (PRRs). Dysbiotic microbiota release pathogen-associated molecular patterns (PAMPs) such as lipopolysaccharide (LPS) and peptidoglycan, which activate Toll-like receptors (TLRs), including TLR2 and TLR4, on mucosal epithelial cells. Upon TLR4 activation, the adaptor protein myeloid differentiation primary response 88 (MyD88) is recruited (Duan T. et al., 2022), triggering the formation of the interleukin-1 receptor-associated kinase (IRAK)/TNF receptor-associated factor 6 (TRAF6) complex. TRAF6 then activates the IκB kinase (IKK) complex, leading to the phosphorylation and degradation of inhibitor of κB (IκB), thereby activating nuclear factor kappa-light-chain-enhancer of activated B cells (NF-κB) and facilitating its translocation into the nucleus (Visioli et al., 2022). Nuclear translocation of NF-κB initiates the transcription of pro-inflammatory genes, upregulating cytokines such as tumor necrosis factor-*α* (TNF-α), interleukin-1β (IL-1β), and interleukin-6 (IL-6) (Wielento et al., 2023). Increased TNF-*α* expression can upregulate transforming growth factor-β (TGF-β) ligands, activating the canonical TGF-β/Smad signaling pathway, where Smad refers to homologs of Sma and Mothers against decapentaplegic. This pathway regulates the phosphorylation of Smad2/3, enabling their complex formation with Smad4 and subsequent nuclear translocation (Liu et al., 2024). It subsequently regulates pro-fibrotic gene expression, induces α-smooth muscle actin (α-SMA), promotes the transdifferentiation of fibroblasts into myofibroblasts, and upregulates the expression of collagen and fibronectin, thereby enhancing extracellular matrix deposition (Shi et al., 2020). Meanwhile, IL-1β can downregulate Smad7 expression, relieving its inhibitory effect on Smad2/3 phosphorylation via competitive binding to the TGF-β receptor, thereby indirectly enhancing TGF-β activity by suppressing this negative feedback mechanism (Bian et al., 2015). TRAF6 activation also regulates the MAP3K-MAP2K-MAPK phosphorylation cascade (Huang et al., 2010), activating downstream signaling molecules such as c-Jun N-terminal kinase (JNK), p38 mitogen-activated protein kinase (p38 MAPK), and extracellular signal-regulated kinase (ERK). This upregulates the expression of tissue inhibitors of metalloproteinases (TIMPs) while inhibiting matrix metalloproteinase (MMP) activity, leading to an imbalance in collagen metabolism and the formation of abnormally cross-linked collagen structures (Tilakaratne et al., 2016). The p38/JNK branch can also activate activator protein-1 (AP-1; composed of c-Fos and c-Jun), further increasing inflammatory factor expression and establishing a positive feedback loop (Clemente et al., 2023). Additionally, ERK can regulate epithelial cell apoptosis, exacerbating mucosal barrier damage. Studies have shown that NF-κB-induced upregulation of cytokines such as TNF-α and IL-1*β* further activates p38 MAPK, while MAPK activation promotes IκBα phosphorylation and facilitates NF-κB nuclear translocation (Zhao et al., 2019). Moreover, MAPK signaling regulates the transcriptional stability of inflammatory mediators through phosphorylation of the RNA-binding protein Tristetraprolin (TTP) (O'neil et al., 2018), indicating a synergistic interaction between NF-κB and MAPK pathways in immune regulation. Furthermore, activation of the NOD-like receptor pyrin domain-containing protein 3 (NLRP3) inflammasome by pathogenic microorganisms induces increased secretion of inflammatory cytokines such as interleukin-1β (IL-1β) and interleukin-18 (IL-18), recruits neutrophils and enhances their phagocytic function, thereby driving mucosal inflammatory responses and amplifying immune defense mechanisms (Cheat et al., 2022).

An imbalance in the T helper 17 (Th17)/regulatory T cell (Treg) equilibrium has been observed during the progression of OSF. Studies indicate that in advanced stages of OSF, the population of Th17 cells and the expression of their associated cytokines are significantly increased, while the number of Treg cells shows a decreasing trend (Liu et al., 2022). Th17 cells activate fibroblasts through the secretion of cytokines such as interleukin-17 (IL-17) and interleukin-21 (IL-21), leading to excessive collagen deposition and promoting both inflammatory responses and fibrotic progression. Conversely, the reduction in Treg cells weakens Treg-mediated immunosuppression. Furthermore, within the inflammatory microenvironment, TGF-β can synergistically promote the differentiation of Th17 cells over Treg cells, thereby exacerbating this imbalance (Lee, 2018). During the malignant transformation period of OSF, a marked increase in Treg cells is observed. Although Th17 cells remain elevated compared to healthy levels, the greater rise in Tregs leads to a significant decrease in the Th17/Treg ratio (Wang et al., 2020; Liu et al., 2022), which facilitates tumor immune escape and is closely associated with tumor progression and poor prognosis. Microbial dysbiosis can disrupt the Th17/Treg balance through multiple pathways. *Porphyromonas gingivalis* infection downregulates the anti-inflammatory cytokine interleukin-10 (IL-10), reducing Treg cell numbers (Li C. et al., 2022), while activating the transcription 3 (STAT3) transcription factor to upregulate the Th17-specific transcription factor retinoic acid receptor-related orphan receptor gamma t (RORγt) and the pro-inflammatory cytokine IL-17A, thereby enhancing Th17 cell responses (Zhang et al., 2021). This directly modulates immune cell differentiation. Furthermore, *Porphyromonas gingivalis* infection reduces indoleamine 2,3-dioxygenase (IDO) activity, leading to tryptophan metabolic imbalance that suppresses Treg differentiation and promotes Th17 polarization (Yang et al., 2022). Thus, the Th17/Treg imbalance induced by microbial dysbiosis plays a critical role in the pathogenesis of OSF.

**Figure 1:**
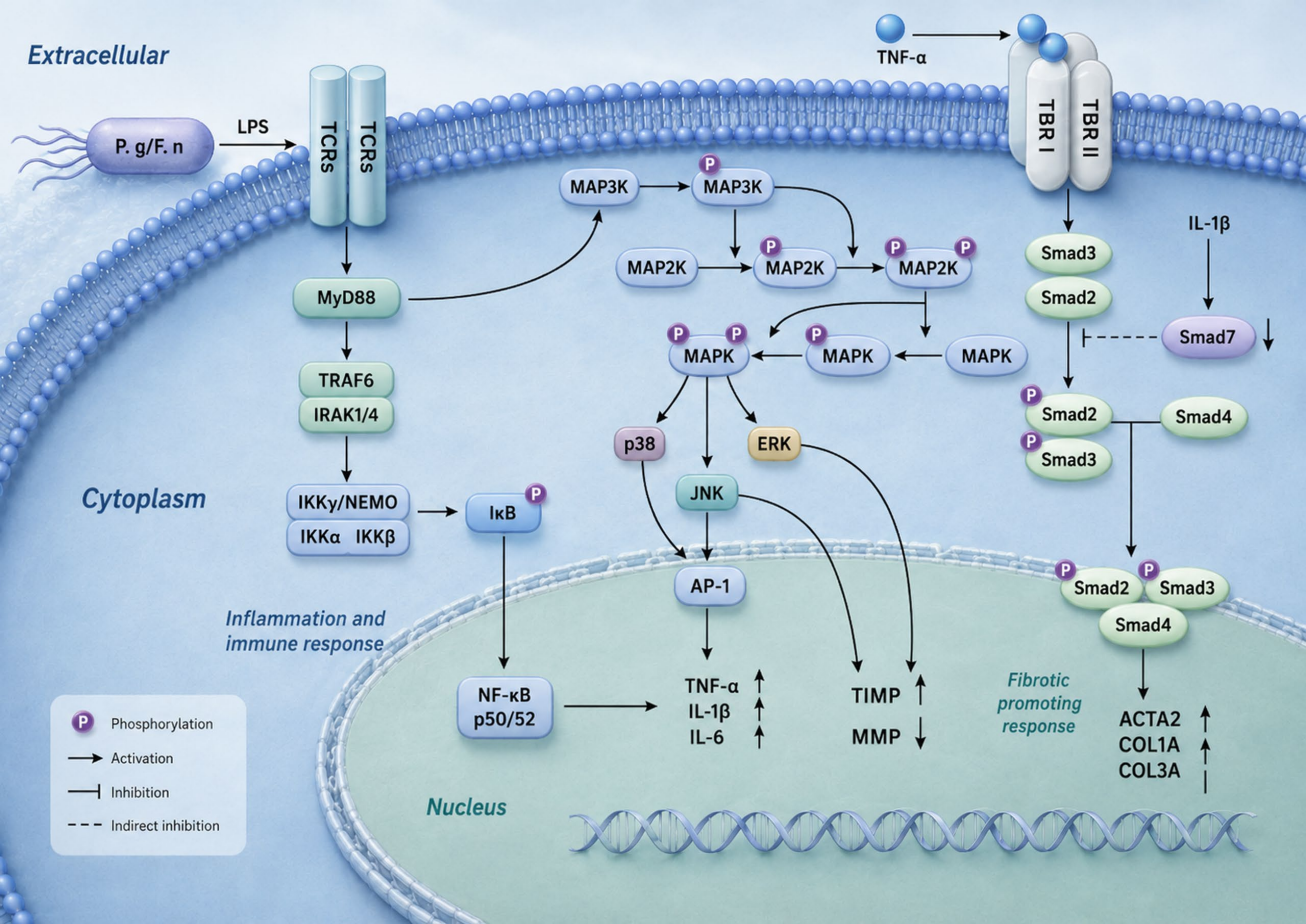
Mechanisms of fibrosis induction mediated by immune alterations in oral microbiota dysbiosis. Created with Figdraw.com.

Dysbiotic microbiota releases lipopolysaccharide (LPS) that activates Toll-like receptors, triggering the formation of the IRAK/TRAF6 complex. This further activates the IKK complex, promoting phosphorylation and degradation of IκB, leading to NF-κB activation and its subsequent nuclear translocation, which increases the transcriptional levels of inflammatory cytokines. Activation of TRAF6 also modulates the MAPK phosphorylation cascade, activating downstream signaling molecules such as JNK, p38 MAPK, and ERK. This upregulates the expression of tissue inhibitors of metalloproteinases while inhibiting the activity of MMP, resulting in an imbalance of collagen metabolism (Figure 1).

### 3.3 Metabolic regulation

Dysbiosis can modulate immune cell differentiation, disrupt inflammatory balance, and compromise the mucosal barrier through microbial metabolites. Tryptophan serves as a precursor for various bioactive compounds and regulates neuronal function, metabolism, and inflammatory responses (Konopelski and Mogilnicka, 2022). Alterations in the microbiota can directly or indirectly influence tryptophan metabolism via the gut-oral axis. Tryptophan is metabolized mainly through the kynurenine, serotonin, and indole pathways, with the indole pathway primarily mediated by gut microbiota (Li, 2023). When microbial composition shifts, metabolism tends toward the kynurenine pathway, leading to reduced production of indole derivatives. As key ligands for the aryl hydrocarbon receptor (AhR), decreased indole compounds result in insufficient AhR activation. AhR activation promotes Treg differentiation and suppresses pro-inflammatory Th17 cells; thus, its impaired activation disrupts the Th17/Treg balance and exacerbates local mucosal inflammation (Romero-Figueroa et al., 2023). Inadequate AhR signaling also reduces IL-22 secretion by innate lymphoid cells, thereby weakening the oral mucosal barrier, impairing epithelial repair, and diminishing antimicrobial peptide expression (Mendes and Schnabl, 2020).

Short-chain fatty acids (SCFAs) also regulate the immune status of the oral mucosa. During dysbiosis, butyrate-producing bacteria (such as members of the order *Clostridiales, Roseburia, and Faecalibacterium*) are significantly reduced (Joubert et al., 2023; Kamer et al., 2024). As major producers of SCFAs, their decreased abundance directly impairs SCFA synthesis (Mehmood et al., 2021). SCFAs can suppress the nuclear translocation of NF-κB via activation of G protein-coupled receptors (GPCRs), thereby reducing the transcriptional activity of inflammatory factors and decreasing the release of pro-inflammatory cytokines such as TNF-*α*, IL-1*β*, IL-6, and IL-8 (Akhtar et al., 2022). GPCR activation also modulates MAPK signaling, inhibiting p38 and ERK1/2 phosphorylation and further limiting cytokine production (Du et al., 2024). Additionally, GPCR activation directly regulates immune cell functions. Activation of GPR43 promotes the differentiation of Foxp3 + Treg cells and enhances their ability to secrete the anti-inflammatory cytokine IL-10 (Zhang L. et al., 2022). It also facilitates the polarization of anti-inflammatory M2 macrophages, suppresses pro-inflammatory M1 macrophage activity, and reduces neutrophil chemotaxis (He and Dong, 2023). A decrease in SCFAs leads to inhibited Treg cell differentiation and an excessive release of inflammatory mediators such as IL-17A and TNF-α (Pandiyan et al., 2019), resulting in uncontrolled inflammatory responses and aggravated tissue damage.

SCFAs also modulate host gene expression through epigenetic mechanisms. Butyrate, by inhibiting histone deacetylase (HDAC) activity, increases histone acetylation levels and opens chromatin structure, thereby enhancing Foxp3 gene expression and promoting Treg cell differentiation (Feng and Xu, 2023). Concurrently, it downregulates Th17-related genes such as RORγt and IL-17, as well as pro-inflammatory factors including IL-6, IL-12, and CXCL10, thus modulating inflammatory responses (Liu X. et al., 2023). Furthermore, studies indicate that HDAC8 inhibition restores H3 K27 acetylation and upregulates PPARγ transcription, ultimately suppressing the expression of TGF-β and its downstream molecules CTGF and α-SMA (Liu Y. et al., 2023b). This suggests that SCFAs-mediated epigenetic regulation via HDAC inhibition may represent another important pathway through which the microbiota influences the progression of OSF.

### 3.4 Oxidative stress

Arecoline-induced oxidative stress is a key mechanism in the pathogenesis of OSF, and a bidirectional interaction exists between oxidative stress and microbial dysbiosis. On one hand, microbial dysbiosis promotes ROS accumulation through both direct bacterial activity and host immune responses. Pathogenic bacteria such as *Porphyromonas gingivalis* and *Fusobacterium nucleatum* exhibit enhanced metabolic activity under dysbiotic conditions, increasing ROS production during anaerobic protein metabolism (Herrero et al., 2016). In particular, cysteine proteases secreted by *P. gingivalis* further aggravate oxidative stress by degrading host proteins and inducing oxygen free radical generation (Al-Attar et al., 2018). Pathogen-associated molecular patterns (PAMPs), especially lipopolysaccharide (LPS), also activate Toll-like receptors on immune cells, leading to pro-inflammatory cytokine production (Li Y. et al., 2022) and NADPH oxidase (NOX)-dependent ROS generation, thereby establishing a positive feedback loop (La Rosa et al., 2020). In addition to enhanced ROS production, dysbiosis also disrupts antioxidant homeostasis. Under dysbiotic conditions, ROS accumulation is further amplified by impaired antioxidant defenses. Inflammatory mediators such as plasma peroxidases neutralize commensal-derived H₂O₂, weakening its antimicrobial activity and promoting pathogen overgrowth (Herrero et al., 2016). In addition, dysbiosis-associated acidification suppresses host antioxidant enzyme activity, further contributing to oxidative imbalance (Zhang Y. et al., 2022).

On the other hand, oxidative stress selectively enriches ROS-resistant microorganisms, thereby enhancing the competitiveness of pathogenic taxa within biofilms through conserved stress-adaptation mechanisms (Yu et al., 2024). The ROS-microbiota relationship is highly individualized and shaped by ecological network structure, with both dominant and low-abundance taxa contributing variably to community responses across individuals (Dzunkova et al., 2018).

Arecoline in betel quid is a key trigger for ROS accumulation. It activates cholinergic receptors in keratinocytes (Chang et al., 2013), triggering Ca^2+^-dependent activation of NOX and subsequent generation of superoxide anions and other ROS (Wang et al., 2024). Arecoline-induced mitochondrial dysfunction impairs electron transport chain activity and ATP production (Mello et al., 2016), promoting electron leakage and further amplifying ROS generation, thereby establishing a self-perpetuating oxidative cycle (Liu et al., 2018). A systematic review indicated that OSF patients exhibit systemic oxidative imbalance, with elevated oxidative stress markers and reduced antioxidant defenses (Saso et al., 2022). The imbalance between oxidants and antioxidants leads to cumulative oxidative damage to cellular macromolecules and functional impairment (Rai et al., 2019).

The persistent oxidative microenvironment established by arecoline exposure and microbial dysbiosis serves as a central driver of OSF progression by promoting fibrotic remodeling and facilitating malignant transformation. TGF-β is a key profibrotic mediator in OSF and exists in a latent complex with latency-associated peptide (LAP). ROS can activate latent TGF-β through several mechanisms, including direct oxidation of LAP (Liu and Desai, 2015), MMP-and plasmin-mediated cleavage of LAP (Costa et al., 2022), and integrin-dependent conformational activation, thereby promoting downstream profibrotic signaling (Robertson and Rifkin, 2013; Liu and Desai, 2015). Activated TGF-β subsequently stimulates the canonical TGF-β/Smad pathway, promoting fibroblast activation, myofibroblast transdifferentiation, and excessive collagen synthesis. ROS further promote extracellular matrix accumulation by TIMPs, thereby suppressing MMP-mediated collagen degradation (Warnakulasuriya et al., 2021). ROS also synergize with copper ions to activate lysyl oxidase (LOX), promoting abnormal collagen cross-linking and insoluble matrix formation (Chaurasia et al., 2019). Furthermore, ROS inhibits prolyl hydroxylase (PHD), leading to stabilization of hypoxia-inducible factor-1α (HIF-1α). HIF-1α cooperates with Notch signaling to upregulate Snail-1, suppress epithelial markers such as E-cadherin, and induce EMT (Chaudhary et al., 2015; Lin et al., 2024). Notably, elevated levels of the DNA damage marker 8-hydroxydeoxyguanosine (8-OHdG) have been observed in the saliva of OSF patients and those with malignant transformation (Nandakumar et al., 2020), suggesting that oxidative stress may contribute to OSF malignant progression via EMT induction and oxidative DNA damage.

**Figure 2:**
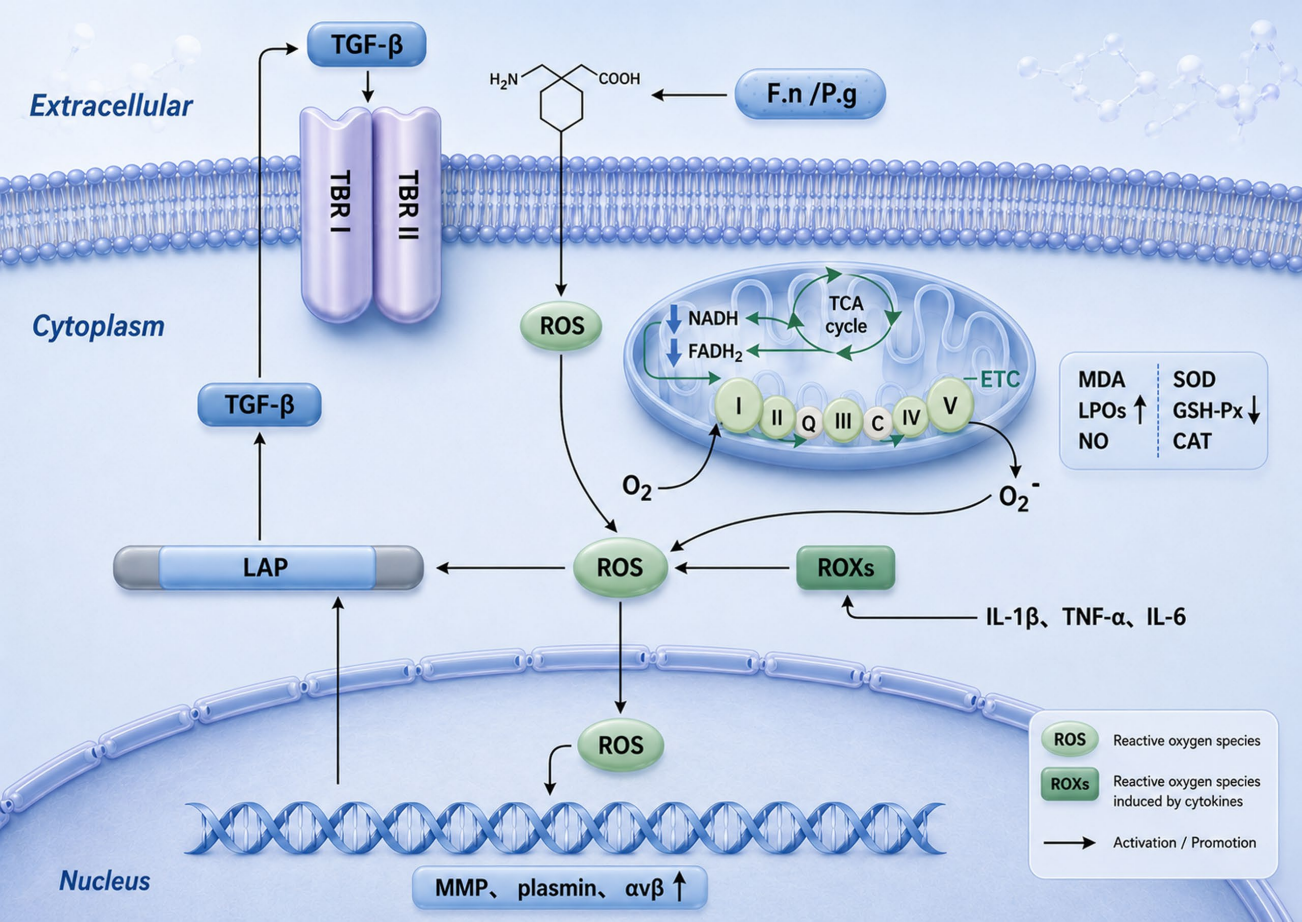
Mechanisms of fibrosis induction mediated by oxidative stress activation in oral microbiota dysbiosis. Created with Figdraw.com.

Under dysbiotic conditions, the anaerobic metabolism of pathogenic bacteria is enhanced, leading to the decomposition of proteins and amino acids and the generation of reactive oxygen species (ROS) that induce oxidative stress. Prolonged oxidative stress results in mitochondrial damage and disruption of the electron transport chain in respiration, during which O₂ is reduced to O₂^−^, further exacerbating ROS accumulation. ROS upregulate the expression of MMPs, plasmin, and *α*v*β*, activating the latent TGF-β precursor LAP and the canonical TGF-β/Smad pathway, thereby regulating the expression of pro-fibrotic genes (Figure 2).

## 4 Potential therapeutic strategies targeting the microbiota

Given the growing evidence linking oral dysbiosis to OSF pathogenesis, modulation of the oral microbiota has emerged as a promising therapeutic approach. Strategies including probiotics, specifically targeted antimicrobial peptides (STAMPs), oral microbiota transplantation (OMT), and dietary modulation have attracted increasing interest because of their potential to restore microbial homeostasis and modulate host-microbiota interactions. It should be noted that current evidence supporting microbiota-targeted interventions in OSF remains limited, with few studies directly evaluating microbiome-modulating therapies in OSF patients or experimental models. Consequently, most proposed strategies are extrapolated from findings in other oral diseases characterized by microbial dysbiosis and chronic inflammation.

### 4.1 Probiotics and targeted antibacterial approaches

Probiotic therapy represents a promising approach for restoring oral microbial balance. Probiotic intervention can reduce oral pathogenic bacterial load, alleviate inflammation, and enhance tissue repair (Lafleur et al., 2023), highlighting its potential in treating OSF through microbiota modulation.

The mechanisms by which probiotic interventions modulate microbial homeostasis include the following aspects:

1. Inhibition of Pathogen Colonization and Growth: Probiotics can occupy receptor sites on the oral mucosa, tooth enamel, or dental plaque, preventing the colonization of pathogens such as *Porphyromonas gingivalis* and *Fusobacterium nucleatum* (Mohd Fuad et al., 2023). They compete with pathogenic bacteria for carbon and nitrogen sources (Homayouni Rad et al., 2023) and secrete bacteriocins and organic acids to restrict their growth (Virk et al., 2024). Additionally, proteases, lipases, and metabolites such as lactic acid produced by probiotics can degrade the biofilm matrix of pathogens, disrupting plaque biofilm structure (Rebelo et al., 2023).
2. Modulation of Host Immune Response: Probiotics such as Lactobacillus and Bifidobacterium suppress the activation of NF-κB, reducing the secretion of inflammatory cytokines like TNF-α and IL-1β to alleviate inflammation. Through Toll-like receptor-mediated signaling, they also induce the secretion of the anti-inflammatory cytokine IL-10, thereby curbing excessive inflammatory responses (Rajasekaran et al., 2024). Furthermore, probiotics stimulate dendritic cells (DCs) to secrete TGF-*β* and IL-10, driving the differentiation of CD4 + Foxp3 + Treg cells, suppressing Th1/Th17 differentiation, and maintaining immune tolerance (Zanetta et al., 2022). Probiotics also promote the differentiation of B cells into plasma cells, enhancing the secretion of sIgA to neutralize pathogens and prevent their invasion. They also activate epithelial cells to release antimicrobial peptides such as β-defensins, strengthening innate immune defense (Fallah et al., 2023).
3. Metabolic Environment Regulation: Prebiotics like xylitol and N-acetylglucosamine can be selectively metabolized by probiotics as carbon sources, promoting probiotic proliferation and inhibiting the energy metabolism of pathogenic bacteria (Mohd Fuad et al., 2023). Short-chain fatty acids (SCFAs) produced by probiotic metabolism help maintain oral redox homeostasis, reducing ROS-induced tissue damage (Virk et al., 2024). Additionally, probiotic interventions can increase the abundance of commensal bacteria, suppress opportunistic pathogens, and restore microbial diversity (Diwan et al., 2023).

Targeted antibacterial therapy represents another effective approach for restoring oral microbial balance. Specifically Targeted Antimicrobial Peptides (STAMPs) represent a key technology for modulating microecological equilibrium. These peptides offer advantages such as reduced risk of bacterial resistance, high thermal stability, potent antimicrobial activity, strong specificity, and minimal toxicity. By conjugating pathogen-specific targeting peptides (e.g., pheromones) with broad-spectrum antimicrobial peptides, STAMPs enable precise eradication of target pathogens (Xu et al., 2025). *In vitro* studies demonstrate that the STAMP significantly reduces the abundance of the cariogenic pathogen *Streptococcus mutans*, showing promising potential for reshaping microbial composition and restoring ecological health (Guo et al., 2015). Additionally, novel antibacterial strategies such as photodynamic therapy (PDT) generate singlet oxygen and free radicals via photosensitizers to disrupt bacterial cell structures. Since its effects are primarily confined to the biofilm surface, PDT causes minimal damage to deeper commensal bacteria, thereby offering selective targeting of pathogenic microorganisms (Tonon et al., 2021).

Despite their promising potential, several challenges remain regarding the therapeutic use of probiotics. The beneficial effects reported in oral diseases are generally modest, and the underlying mechanisms remain incompletely understood, particularly regarding their ability to achieve stable colonization within the oral cavity (Hoare et al., 2017). In addition, current evidence is limited by small sample sizes and a lack of high-quality clinical studies. The long-term efficacy of probiotics may also be influenced by persistent host- and environment-related risk factors, while challenges related to strain safety, formulation stability, and clinical translation remain to be addressed.

### 4.2 Microbiota transplantation and dietary modulation

Oral Microbiota Transplantation (OMT) is an emerging microbial therapy aimed at restoring the ecological balance of the oral microbiota by transferring microbial communities from healthy donors to patients. Preliminary animal studies have shown that OMT can reduce the levels of IL-1, IL-6, TNF-*α*, and TGF-β in oral mucosal tissues, thereby alleviating mucosal damage (Xiao et al., 2021). From a microbiological perspective, OMT reversed the enrichment of pathogenic bacteria under inflammatory conditions and increased the α-diversity of the oral microbiota (Xiao et al., 2021). Despite its promising therapeutic potential in animal models, the complexity and individual variability of human oral microbiota make the efficacy of OMT difficult to predict and standardize (Li et al., 2024). Furthermore, the lack of large-scale, multi-center clinical trials to validate its efficacy and safety, along with the absence of standardized protocols and donor screening criteria (Lindo et al., 2024), currently limits the clinical application of OMT.

Dietary modification serves as another important approach to modulating the oral microbiota. On one hand, dietary interventions can alter microbial composition by changing the nutrient substrates available in the oral cavity. Different types of diets provide varying carbon sources, thereby influencing microbial metabolic activities. For instance, short-chain fatty acid (SCFA)-producing bacteria thrive on fermentable fibers, meaning a high-fiber diet can promote the growth of such bacteria (Yap and Marino, 2018), contributing to oral mucosal immune regulation. On the other hand, dietary interventions can influence microbial structure by altering the physicochemical properties of the oral environment. A low-carbohydrate diet reduces substrates for microbial fermentation, thereby preventing the suppression of acid-sensitive probiotic bacteria in an acidic environment and helping maintain microbial diversity (Giordano-Kelhoffer et al., 2022). Additionally, nitrate-rich foods such as leafy greens promote the reduction of nitrate to nitrite by nitrate-reducing bacteria, which can be further converted to ammonia to neutralize acidic conditions and enhance microbial stability (Rosier et al., 2020). Moreover, polyphenol-rich foods like tea can inhibit the growth of pathogenic bacteria and disrupt plaque biofilm structure. Studies have shown that polyphenols exert antibacterial effects against periodontal pathogens such as *P.gingivalis* and *F.nucleatum* by disrupting cell membranes or inhibiting virulence factor expression (Mashal et al., 2024).

While STAMPs offer the advantage of selectively targeting pathogenic microorganisms while preserving beneficial commensals, several challenges remain. The complexity of the oral microbial ecosystem makes it difficult to identify and target specific disease-associated microorganisms (Elashiry et al., 2021), and suppression of individual pathogens alone may be insufficient to restore long-term microbial homeostasis (Sheets et al., 2008). Moreover, persistent host- and environment-related risk factors may compromise therapeutic efficacy, while issues regarding delivery efficiency, long-term safety, and clinical translation require further investigation (Table 2).

**Table 2:** Potential therapeutic strategies based on microbiota modulation.

Intervention strategy	Specific approach	Core mechanisms/advantages	Key outcomes	References
Probiotic therapy	Suppression of Pathogens	• Colonization competition • Bacteriocin and organic acid production • Biofilm disruption	• Pathogen suppression • Plaque disruption	Mohd Fuad et al. (2023) Homayouni Rad et al. (2023) Virk et al. (2024) Rebelo et al. (2023)
	Host Immune Modulation	• NF-κB inhibition • Treg induction • sIgA production	•Reduced pro-inflammatory cytokines •Immune tolerance	Rajasekaran et al. (2024) Zanetta et al. (2022) Fallah et al. (2023)
	Metabolic regulation	• SCFA production; • Prebiotic-mediated selective • Proliferation	• Redox homeostasis • Restored microbial diversity	Mohd Fuad et al. (2023) Virk et al. (2024) Diwan et al. (2023)
Targeted antimicrobials	Specifically targeted antimicrobial peptides (STAMPs)	• Targeted pathogen recognition • Selective antimicrobial activity	• High specificity • Low toxicity • Reduced resistance	Xu et al. (2025) Guo et al. (2015)
	Photodynamic Therapy (PDT)	• ROS-mediated bacterial killing	• Selective pathogen elimination • Microbiota preservation	Tonon et al. (2021)
Microbiota transplantation	Oral microbiota transplantation (OMT)	•Healthy microbiota transplantation •Reverses pathogen enrichment	• Reduced inflammation; • Increased microbial diversity	Xiao et al. (2021) Li et al. (2024) Lindo et al. (2024)
Dietary modulation	High-fiber diet	• SCFA production • Enrichment of SCFA-producing bacteria	• Enhanced mucosal immunity	Yap and Marino (2018) Giordano-Kelhoffer et al. (2022) Rosier et al. (2020) Mashal et al. (2024)
	Low-carbohydrate diet	• Reduced acidogenic substrates • pH maintenance	• Preserved commensals; • Maintained microbial diversity	
	Nitrate-rich diet	• Nitrate reduction • pH buffering	• Enhanced microbial stability	
	Polyphenol-rich diet	• Membrane disruption • Virulence inhibition	• Pathogen suppressio; • Biofilm disruption	

### 4.3 Comparison with conventional therapies

Current management of OSF primarily relies on habit cessation, corticosteroids, antioxidants, hyaluronidase, physiotherapy, and surgical intervention in advanced cases (Qin et al., 2023). These approaches mainly target inflammation, fibrosis, and symptom relief, and can provide short-term clinical benefits. However, their ability to prevent disease progression or malignant transformation remains limited. Compared with conventional therapies, microbiota-based interventions offer a distinct therapeutic concept by targeting microbial dysbiosis and host-microbiota interactions, which have been increasingly associated with OSF progression and disease-related inflammatory responses. Whereas current treatments primarily focus on controlling inflammation, reducing fibrosis, and alleviating symptoms, modulation of the oral microbiota may provide opportunities to address upstream disease-driving mechanisms and promote long-term restoration of oral ecological homeostasis (Dong et al., 2022). In addition, microbiota-based approaches may avoid some of the adverse effects associated with prolonged pharmacological therapy or invasive surgical procedures (Chen et al., 2025). Despite these potential advantages, as highlighted in the preceding sections, microbiota-targeted therapies remain largely experimental, unlike conventional treatments that have already been incorporated into clinical practice. The efficacy, safety, optimal delivery methods, and long-term outcomes have yet to be established, particularly in the context of OSF, where direct clinical and preclinical evidence remains scarce.

Notably, conventional therapies may also influence the oral microbiota. For example, corticosteroids can alter local immune responses and mucosal ecological conditions, potentially affecting microbial composition (Sedghi et al., 2021). However, the impact of these treatments on the oral microbiome in OSF has not yet been systematically evaluated.

## 5 Conclusion

### 5.1 Summary

Oral microbiota dysbiosis refers to multifactorial and multidimensional alterations in the native microbial composition of the oral cavity, characterized by the replacement of mutualistic commensals by potential pathogenic microorganisms (Min et al., 2023). The interaction between the host and the altered microbiota plays a critical role in the pathogenesis and progression of OSF. This review systematically summarizes the pathological relationship between microbial dysbiosis and OSF, the core mechanisms of host-microbiota interactions, and advances in potential therapeutic strategies. Areca nut-derived alkaloids and other chemical components induce dysbiosis by altering the oral microenvironment, leading to enrichment of pathogenic bacteria and depletion of beneficial species. The dysbiotic microbiota promotes fibrotic progression through mechanisms including disruption of the mucosal barrier, activation of immune-inflammatory responses, and induction of metabolic dysregulation. Microbiota-targeted strategies such as probiotics, targeted antibacterial therapy, and microbiota transplantation have demonstrated promising potential for clinical application.

### 5.2 Current limitations

Despite the growing evidence linking oral microbiota dysbiosis to OSF, several important limitations and unresolved questions remain. On one hand, while existing studies indicate that OSMF is primarily associated with chemical stimuli such as areca nut chewing, it remains unclear whether microbial dysbiosis is an independent pathogenic factor or a consequence of mucosal damage. Longitudinal studies comparing microbial dynamics between early and late stages of OSF are needed to clarify the temporal relationship between dysbiosis and OSF onset (Tilakaratne et al., 2016). On the other hand, a specific pathogenic bacterial signature for OSF has not yet been established (Willis and Gabaldon, 2020). Metagenomic approaches are required to identify OSF-associated bacterial strains and to correlate microbial profiles with clinical stages for early diagnostic potential. Whether microbial metabolites amplify the abnormal expression of pro-fibrotic genes through epigenetic regulation also warrants further investigation (Tilakaratne et al., 2016). Multi-omics integration of microbiome and host data is essential to elucidate the synergistic mechanisms between dysbiosis and genetic susceptibility. Furthermore, microbiota-targeted therapeutic strategies remain largely experimental, and their efficacy, safety, optimal administration, and long-term outcomes in OSF have yet to be established. Extensive *in vitro*, animal, and clinical studies are required before these approaches can be translated into clinical practice.

### 5.3 Future perspectives

Addressing the current limitations and unresolved questions will be essential for advancing microbiota-based research and its clinical application in OSF. Clarifying the causal relationship between microbial dysbiosis and OSF through longitudinal and mechanistic studies should be a priority. Integrating multi-omics approaches may further facilitate the systematic identification of key nodes within the microbe–host interaction network, while identifying reproducible microbial signatures and functional microbial metabolites associated with different stages of OSF may improve early diagnosis and reveal novel therapeutic targets. In addition, well-designed preclinical and clinical studies are needed to evaluate the efficacy, safety, and long-term outcomes of microbiota-targeted interventions. These efforts may also support the development of OSF-specific probiotic formulations and personalized microbiome-based therapies, thereby promoting their clinical translation.
